# Comparison of Spinal Cord Regeneration Capacity in Zebrafish and Medaka

**DOI:** 10.1007/s11064-025-04389-9

**Published:** 2025-04-25

**Authors:** Shun Aoki, Masato Hori, Hanjie Zhang, Hiroshi Tsujioka, Toshihide Yamashita

**Affiliations:** 1 Department of Molecular Neuroscience, Graduate School of Frontier Biosciences, Osaka University, 2-2, Yamadaoka, Suita, Osaka Japan; 2 Department of Molecular Neuroscience, Graduate School of Medicine, Osaka University, 2-2, Yamadaoka, Suita, Osaka Japan; 3 WPI Immunology Frontier Research Center, Osaka University, 2-2, Yamadaoka, Suita, Osaka Japan; 4 Department of Neuro-Medical Science, Graduate School of Medicine, Osaka University, 2-2, Yamadaoka, Suita, Osaka Japan

**Keywords:** Spinal cord regeneration, Zebrafish, Medaka, Cross-species comparison, RNA-seq

## Abstract

In mammals, spinal cord injury often results in permanent impairment of motor function owing to ineffective tissue regeneration. Unlike mammals, zebrafish have the remarkable ability to regenerate many tissues, including the spinal cord. Cross-species comparison is an attractive approach for revealing regeneration-specific mechanisms, but the large evolutionary distance between species sometimes hinders direct comparison. Recent studies have revealed that another model fish species, medaka, has a low regenerative ability in some tissues, making comparisons with them advantageous to revealing regeneration-specific mechanisms. However, their spinal cord regenerative ability has not been compared to other models. In this study, we functionally and histologically compared the spinal cord regeneration abilities of zebrafish and medaka. Swimming speed recovery was significantly lower in medaka than in zebrafish. Bridging of glia and neural tissue were thinner in medaka than in zebrafish. Axonal extension across the injured site was observed in zebrafish but not in medaka. Comparison of their gene expression profiles revealed genes involved in “Regeneration” were upregulated in zebrafish, whereas genes related to “Synaptic signaling” were downregulated in medaka. These results suggest that the ability to regenerate the spinal cord is lower in medaka than in zebrafish, making medaka an attractive model for revealing the mechanisms of spinal cord regeneration.

**Supplementary Information:**

The online version contains supplementary material available at 10.1007/s11064-025-04389-9.

## Introduction

Spinal cord injury (SCI) affects more than 2.5 million patients worldwide [1]. The major cause of SCI is traumatic damage caused by accidents, such as traffic accidents or falls. Many complications also threaten patients’ quality of life. There is no effective treatment except rehabilitation, which only slightly improves the recovery of motor function.

The pathology of SCI has been previously studied in animal models. In mice, a scar forms at the lesion site after SCI [2]. The scar is comprised of stromal-derived fibroblasts, inflammatory immune cells, and hypertrophic astrocytes. It blocks axonal regeneration both mechanically and chemically, resulting in the failure of functional recovery after SCI.

In contrast, zebrafish have a remarkable ability to regenerate injured spinal cords. In zebrafish, immune cell infiltration is observed during the early phase. The immune system and inflammation play an important role in the regeneration of the central nervous system [3]. At the early phase, interleukin-1β plays a crucial role in axonal regeneration [4]. Additionally, tumor necrosis factor-α secreted by macrophages and neurotrophin 3 derived from regulatory T cells promote neurogenesis in zebrafish [5, 6]. The immune cells potentiate axonal regeneration via rapid clearance of myelin debris [7]. Glial bridging and axonal regeneration then follow. Glial bridges are formed by ependymoradial glia, which share features with radial glia and ependymal cells [8]. Although the contribution of glial bridges is controversial [9–11], they are thought to serve as scaffolds for axons to regrow across the injured site. Finally, the injured spinal cord is remodeled into an almost normal structure. The morphology of the central canal is restored and the injured site is almost indistinguishable from the intact spinal cord [12]. Morphological regeneration is accompanied by prominent functional recovery. Swimming capacity is severely affected after SCI. One week after SCI, the swimming distance of zebrafish significantly decreases. However, the swimming ability of zebrafish spontaneously recovers after approximately six weeks [13]. Understanding the molecular mechanisms that underlie their high regenerative ability would be helpful in developing novel therapeutic approaches for SCI.

Although comparative analyses of tissue regeneration between zebrafish and mice are potentially effective in revealing the factors that determine their regenerative abilities, the large evolutionary distance between them sometimes makes direct comparisons difficult. Medaka is another model fish with advantages for genetic analyses such as a completely sequenced reference genome, short generation time, and a well-established efficient genome editing method [14]. Medaka shares many biological features with zebrafish, including size, diet, organ system, gross anatomy, and living environment. Surprisingly, recent studies have found that unlike zebrafish, which regenerate many organs upon experimental injury, medaka show lower regenerative capacity in some tissues. For example, the capacity of medaka to regenerate the heart or retina is lower than that of zebrafish [15–19]. Comparative analyses of these species, which show remarkably distinct regenerative abilities with shared biological features, are advantageous for revealing regeneration-specific molecular mechanisms. However, the ability of zebrafish and medaka to regenerate the spinal cord has not yet been compared.

Here, we first examined whether swimming ability is recovered in adult medaka after complete transection of the spinal cord and found that the recovery of swimming ability was significantly lower in medaka than in zebrafish. We performed immunostaining to assess the remodeling processes in zebrafish and medaka and found that the glial bridges and neuronal tissue were significantly thinner in medaka than in zebrafish. We labeled long projecting axons using an axonal tracer and did not find any apparent axonal extension beyond the lesion in medaka. Finally, we evaluated the gene expression profiles of injured zebrafish and medaka spinal cords. Genes involved in regeneration were upregulated in zebrafish, whereas those involved in synapses were downregulated in medaka after SCI. Many previous studies have focused on the spinal cord regeneration capacity of zebrafish (including gene expression analysis); however, no studies have compared the spinal cord regeneration capacity of closely related species. Therefore, this is the first study to provide gene expression profiles of zebrafish and medaka, which are closely related species with low spinal cord regeneration capacity, to understand why zebrafish have high spinal cord regeneration capacity.

## Materials and methods

### Animals

AB Zebrafish (*Danio rerio*) were obtained from the Center of Medical Innovation and Translational Research (CoMIT), Osaka University. OK-Cab Medaka (*Oryzias latipes*) were obtained from the National Bioresource Project (NBRP) Medaka (strain ID: MT830). Both male and female adult fish (3–15 months old) were used in this study and maintained in a constant ambient temperature (24–25 °C) under a 14 h light/10 hr dark cycle in CoMIT. All experiments followed a protocol approved by the Institutional Animal Care and Use Committee at Osaka University (permission number 01-014-010).

### Spinal Cord Injury (SCI)

Surgical procedures were performed as previously described by another group [20]. Fish were anesthetized with MS-222 (0.02%, Sigma), and Vannas spring scissors were used to make a small incision that completely transected the spinal cord between the 15th and 16th vertebrae. The total number of vertebrae is 32 in zebrafish and 27–30 in medaka [21, 22]. Therefore, the transected spinal cord levels were approximately midpoint between rostral-most and caudal-most vertebrae in both species. Complete transection was confirmed visually during surgery. Following surgery, fish recovered from anesthesia in water containing gentamicin (10 mg/L). In the sham surgery group, fish were anesthetized with MS-222, their skin and muscle were incised with a scalpel, and they recovered from the anesthesia as described above.

### Behavioral Tests

Behavioral tests were performed as previously described by another group, with some modifications [23]. We used five to six fish for each group (medaka_sham group, medaka_SCI gropup zebrafish_sham group and zebrafish_SCI group). Five to six fish (6–9 months old) were placed in a transparent water tank (20 × 20 × 20 cm, 15 cm water depth) and allowed to swim freely. After habituation for at least 15 min, a video was recorded for 10 min. The tank was set on a transparent acrylic plate and a tilted mirror was placed under it, allowing simultaneous observation of the front and bottom views (Supplementary Fig. 1a).

At week 0, following the behavioral test, SCI was performed, and behavioral tests were performed once a week until 8 weeks post injury (wpi).

**Fig. 1:**
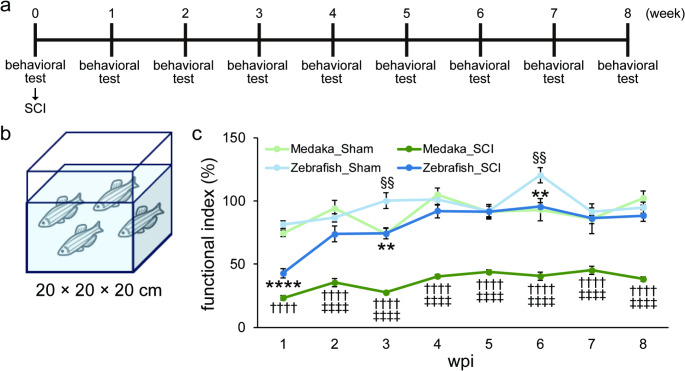
Medaka show lower functional recovery after SCI than zebrafish. (**a**) Experimental time course for the behavioral test and SCI. (**b**) Tank used in behavioral test. (**c**) Average speed in behavioral test. Light green indicates medaka with sham surgery, dark green indicates medaka with SCI, light blue indicates zebrafish with sham surgery, and dark blue indicates zebrafish with SCI. The vertical axis represents the functional index, with the value of the speed before surgery set as 100%. The horizontal axis represents the wpi. The statistical results of the main comparisons are presented. The statistical results for comparisons between all groups are provided in Supplementary Table 3. Mean ± SEM. *n* = 5–6, ** *P* < 0.01, **** *P* < 0.0001 (vs. zebrafish with Sham surgery), ††††*P* < 0.0001 (vs. medaka with Sham surgery), ‡‡‡‡*P* < 0.0001 (vs. zebrafish with SCI), §§*P* < 0.01 (vs. medaka with Sham surgery), two-way ANOVA with Sidak’s multiple comparisons test. *P* < 0.0001 (time), *P* < 0.0001 (condition) *P* = 0.0003 (interaction), SCI, spinal cord injury; SEM, standard error of the mean; wpi, weeks post injury

The 3D location of the animal was identified from tracked images of the front and bottom views using idTracker [24]. The speed of the fish at each frame was then calculated. The functional index was calculated with the average speed of each group before SCI set as 100% (Fig. 1c). The relative speed was calculated with the average speed of sham group set as 100% (Supplementary Fig. 1d).

### Immunohistochemistry (IHC)

**Fig. 2:**
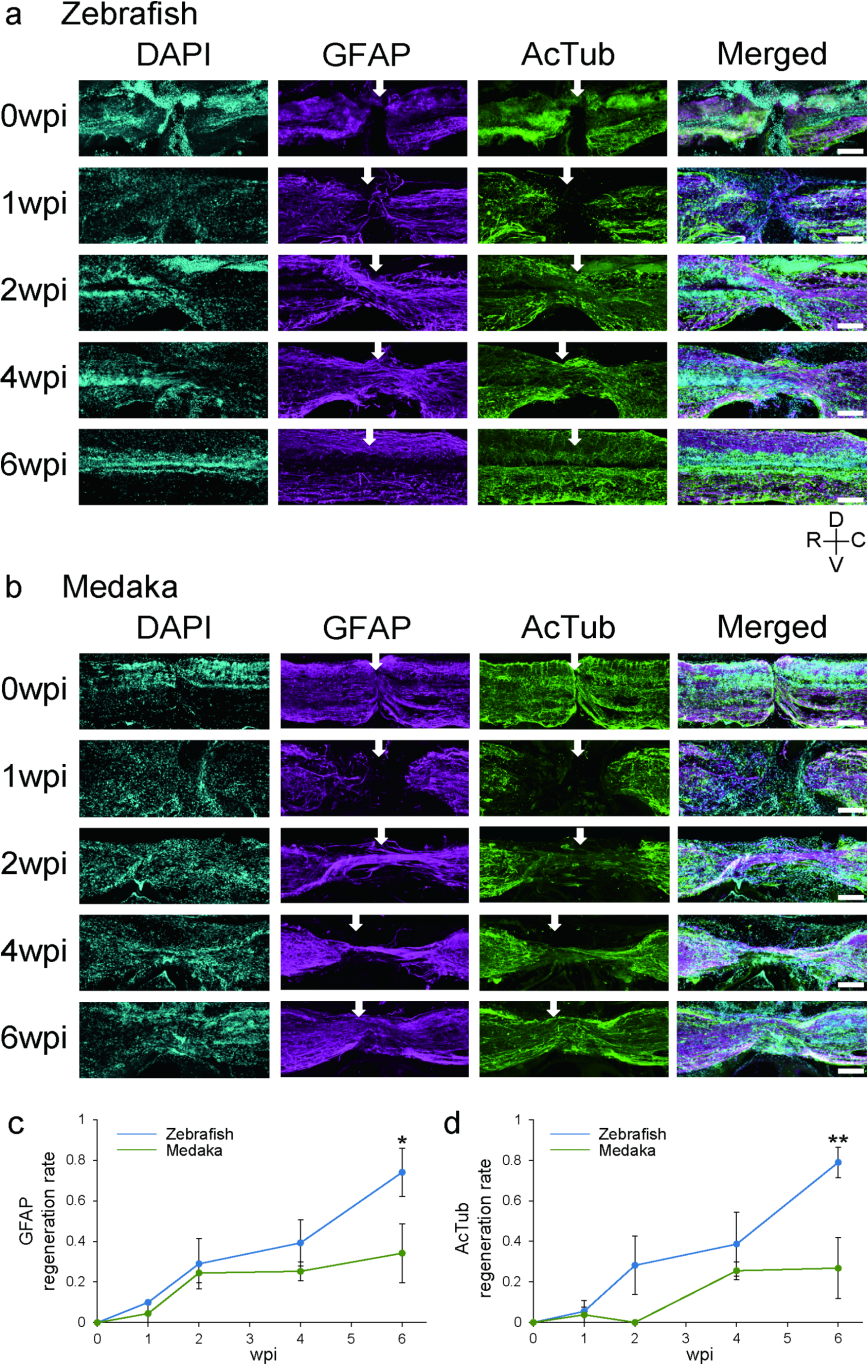
Glial bridges and axonal bundles formed in medaka are thinner than those in zebrafish after SCI. (**a**, **b**) Sagittal section of lesion site labeled with anti-GFAP or anti-AcTub antibody, DAPI, or merged images in zebrafish (**a**) or medaka (**b**) at 0, 1, 2, 4, 6 wpi are shown. D, dorsal; V, ventral; R, rostral; C, caudal. Scale bars: 100 μm. The white arrows indicate the location measured to assess the regeneration rate. (**c**, **d**) Regeneration rate of GFAP (**c**) or AcTub (**d**) at 0, 1, 2, 4, 6 wpi. The vertical axes represent the regeneration rate. The horizontal axes represent the wpi. Blue indicates zebrafish and green indicates medaka. Mean ± SEM. *n* = 3, * *P* < 0.05, ** *P* < 0.01, Sidak’s multiple comparisons test. AcTub, acetylated tubulin; DAPI, 4′,6-diamidino-2-phenylindole; GFAP, glial fibrillary acidic protein; SCI, spinal cord injury; SEM, standard error of the mean; wpi, weeks post injury

IHC was performed as previously described by another group with some modifications [20]. Three fish (3–15 months old) of each species were euthanized at each point (0 wpi, 1 wpi, 2 wpi, 4 wpi and 6 wpi) and fixed in Davidson solution (0.7% formaldehyde, 35% ethanol, 10% acetic acid) overnight at 4 °C and washed with ethanol. After immersion in 30% sucrose / 0.1 M phosphate buffer, fish were embedded in a 25% gelatin, 15% sucrose solution on dry ice and stored at -80 °C. Sagittal Sect. (20-µm thick) were prepared on a cryostat and mounted on glass slides. After washing with phosphate buffered saline (PBS), the sections were blocked with blocking buffer (PBS, 0.3% Triton X-100, and 3% bovine serum albumin) for 1 h at RT. Subsequently, the sections were incubated with primary antibody in blocking buffer overnight at 4 °C. The primary antibodies used were a 1:800 dilution of mouse anti-acetylated tubulin (AcTub) antibody (Sigma, T6793-100UL) and a 1:2 dilution of rabbit anti-glial fibrillary acidic protein (GFAP) (Dako, IR524). Although the anti-GFAP antibody is a ready-to-use antibody, we found that it also works with 1:2 dilution. After washing with PBS, the sections were incubated with secondary antibodies in blocking solution for 1 h at room temperature in the dark. The secondary antibodies used were 4 µg/ml of Alexa Flour 568-conjugated goat anti-mouse IgG (Invitrogen) and 4 µg/ml of Alexa Flour 488-conjugated goat anti-rabbit IgG (Invitrogen). DAPI (4′,6-diamidino-2-phenylindole; 1 µg/ml) was applied with the secondary antibodies. The sections were then washed with PBS and mounted using fluorescence mounting medium (Dako). All images were acquired using a confocal laser-scanning microscope (FV3000, Olympus, Fig. 2) or the fluorescent microscope (BX53, Olympus, Supplementary Fig. 3). Dorsoventral thickness of the lesion was measured using images labeled with anti-GFAP or anti-AcTub antibodies (Supplementary Fig. 2a). The regeneration rate was normalized to the average thickness at the site rostral and caudal to the lesion (Supplementary Fig. 2a).

### Axonal Tracing

Axonal tracing was performed as previously described by another group, with some modifications [25]. Tetramethyl rhodamine dextran amine (RDA, 10%; Invitrogen, D1817) was dissolved in distilled water and stored at -80 °C. The RDA stock solution was air-dried on pieces of Parafilm to obtain crystals, which were then used for tracer application to the spinal cord. Three to eleven Fish (3–5 months old) were anesthetized with MS-222 (0.02%), a very small spinal cord tissue was removed 4 mm rostral to the SCI site, and RDA crystals were applied there. Following surgery, the fish recovered from anesthesia in water containing gentamicin (10 mg/L). Forty-eight hours later, the fish were euthanized and fixed in 4% paraformaldehyde. After washing with PBS, the fish were made transparent using CUBIC (Clear, Unobstructed Brain/Body Imaging Cocktails and Computational analysis, Tokyo Chemical Industry) [26]. All images were acquired by adjusting the laser power and HV to minimize background noise and to observe signal in the rostral axons at the similar level across samples using a confocal laser-scanning microscope (FV3000).

### RNA-Seq Data Analysis

We collected spinal cords from one fish (3–10 months old) for each biological replicate for RNA-seq. The sample was not pooled. The number of biological replicates was 3 without technical replicate. Spinal cord tissue 1-mm wide (from 500 μm rostral to 500 μm caudal to the lesion) was collected at 2 wpi from three samples, with each sample representing one fish. Spinal cord tissue collected at the same level from non-injured fish was used as a control. RNA was extracted from the tissues using RNeasy micro kit (Qiagen, 74104) with DNase treatment using RNase-Free DNase Set (Qiagen, 79254). A library for sequencing was constructed from the amplified total RNA. A total of 51–88 million 100-bp paired reads/sample were produced using DNBSEQ-G400RS (MGI Tech). The reads were mapped to the zebrafish genome GRCz11 and medaka genome HdrR109 using STAR [27]. Read counts were obtained using HTSeq [28] with Danio_rerio.GRCz11.109.gtf or Oryzias_latipes.ASM223467v1.109.gtf [27] as the reference gene model. The following analyses were performed using R software [29] with the appropriate packages. Statistical tests for differentially expressed genes were performed, and normalized read counts were obtained using DESeq2 [30]. Gene Ontology (GO) enrichment analysis of the differentially expressed genes was performed using ShinyGO v0.80 [31].

### Statistical Analysis

All data are presented as mean ± standard error of the mean (SEM), and statistical significance was set at *P* < 0.05 or adjusted *P* < 0.05. An unpaired two-sided Student’s t-test was used for comparisons between two groups, and two-way analysis of variance (ANOVA) with post-hoc Sidak test was used for multiple groups. Statistical analyses were conducted using GraphPad Prism 7 (GraphPad Software). For RNA-seq experiments, Wald test was used.

## Results

### Medaka Have Lower Ability To Recover Motor Function than Zebrafish after SCI

To compare functional motor recovery between zebrafish and medaka after SCI, we evaluated their swimming distances for 10 min ad libitum (Fig. 1a, b, Supplementary Movies 1–6). Swimming speed was not significantly different between zebrafish and medaka before surgery (Supplementary Fig. 1b). We calculated and compared the functional index, taking the value before surgery as 100% for each species.

In the analysis normalized by the values before surgery, Sham surgery slightly decreased functional index at 1wpi (approximately 70–80% compared with pre-surgery), but there was no significant difference between the sham groups of zebrafish and medaka. Subsequently, the functional indexes in both sham groups kept at high level (Fig. 1c). In zebrafish, SCI significantly decreased functional index at 1 wpi (approximately 40% compared with pre-surgery) (Fig. 1c). Subsequently, it gradually increased and almost reached the functional index of the sham group at 6 wpi or later. In medaka, SCI significantly decreased functional index at 1 wpi (approximately 20%), suggesting that spinal cord transection impairs the free swimming function also in medaka. Surprisingly, unlike zebrafish, the functional index remained almost the same even at 8 wpi (Fig. 1c), resulting in a significant difference between zebrafish and medaka at 2–8 wpi, suggesting that the ability to recover the free swimming function is lower in medaka than in zebrafish. The actual swimming distance or swimming distance normalized to sham produced the similar result (Supplementary Fig. 1c, d).

### Remodeling Processes Differ in Medaka and Zebrafish after SCI

Next, to compare histological changes at the injured site in the spinal cord, we labeled ependymoradial glia or neurons with anti-GFAP or anti-AcTub antibodies, respectively, which were previously used to label cells in zebrafish [20, 32]. We observed a clear signal in the spinal cord when these antibodies were applied, and no signal was detected when no primary antibodies were applied in IHC in either zebrafish or medaka, suggesting that these antibodies labeled ependymoradial glia and neurons in both species (Supplementary Fig. 2b). In zebrafish, complete transection was confirmed immediately after injury (0 wpi, Fig. 2a). Glial and axonal bridging was observed at 2 wpi, which is consistent with the results of a previous study [2]. The bridged tissue gradually became thicker and was almost indistinguishable from the intact tissue at 6 wpi (Fig. 2a, Supplementary Fig. 2c, 3), which was consistent with a previous study [20, 33]. In medaka, we confirmed the complete transection of glia and neurons at 0 wpi and observed glial and axonal bridging at 2 wpi (Fig. 2b). However, unlike zebrafish, the bridged tissue did not become as thick as the intact tissue even at 6 wpi (Fig. 2b, Supplementary Fig. 2c, 3).

To quantitatively evaluate the IHC results, GFAP and AcTub regeneration rates were calculated from the obtained images (Fig. 2c, d). At 6 wpi, the GFAP and AcTub regeneration rates of zebrafish were approximately 0.79 and 0.75, respectively, whereas those of medaka were approximately 0.34 and 0.27, respectively, both of which were significantly different. These results suggest that medaka have a lower ability to regenerate glia and neural tissue during the remodeling process after SCI than zebrafish.

### Axonal Regeneration across Completely Transected Spinal Cord Is Observed in Zebrafish, but not in Medaka

**Fig. 3:**
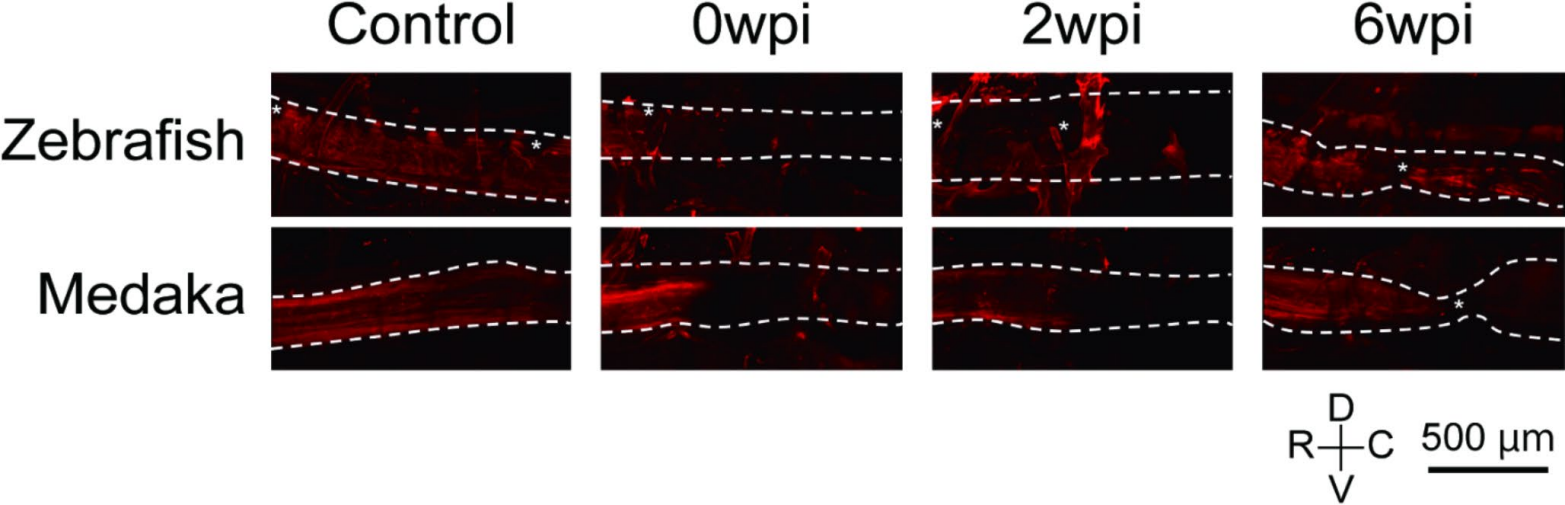
Axon outgrowth across lesion was observed in zebrafish but not observed clearly in medaka 6 weeks after SCI. Representative images of the sagittal section of the lesion labeled with an axonal tracer. Dashed lines indicate the spinal cord. * No signal was observed, possibly because of the melanin pigment. D, dorsal; V, ventral; R, rostral; C, Caudal. Scale bar: 500 μm

To assess the outgrowth of injured long projecting axons more clearly, we placed an axonal tracer 4 mm rostral to the lesion to observe the outgrowth of severed axons across the completely transected spinal cord (Fig. 3). No signal was observed in some areas (shown as *), possibly because of melanin pigment deposition, which had little effect on this observation. Labeled axons were observed rostral to the lesion, but the signal was completely lost at the lesion, confirming complete axonal severance in both zebrafish and medaka at 0 wpi (Fig. 3). In zebrafish, labeled axons passed through the lesion, and the signal was observed at about 500 μm caudal to the lesion at 6 wpi, which is consistent to a previous study [20] (Fig. 3, Supplementary Fig. 4). In contrast, in medaka, signals of labeled axons were only detected rostral to the lesion and did not cross there at 6 wpi (Fig. 3, Supplementary Fig. 4). These results suggest that the ability to regenerate severed axons across the transected spinal cord is limited in medaka compared to zebrafish.

### Gene Expression Profile in the SCI Site Differs between Zebrafish and Medaka

Recovery of locomotor function began at 2 wpi (Fig. 1). Moreover, some axonal and glial processes extended beyond the SCI site by 15 wpi [2]. Consistent with a previous report, we observed glial and axonal bridging at 2 wpi in zebrafish (Fig. 2). Therefore, we considered that 2 weeks after SCI is a key point at which factors involved in functional/histological recovery increase/decrease. We compared the changes in gene expression in injured spinal cord tissue at 2 wpi to clarify the possible causal factors that determine the differences in regenerative capacity between zebrafish and medaka.

**Fig. 4:**
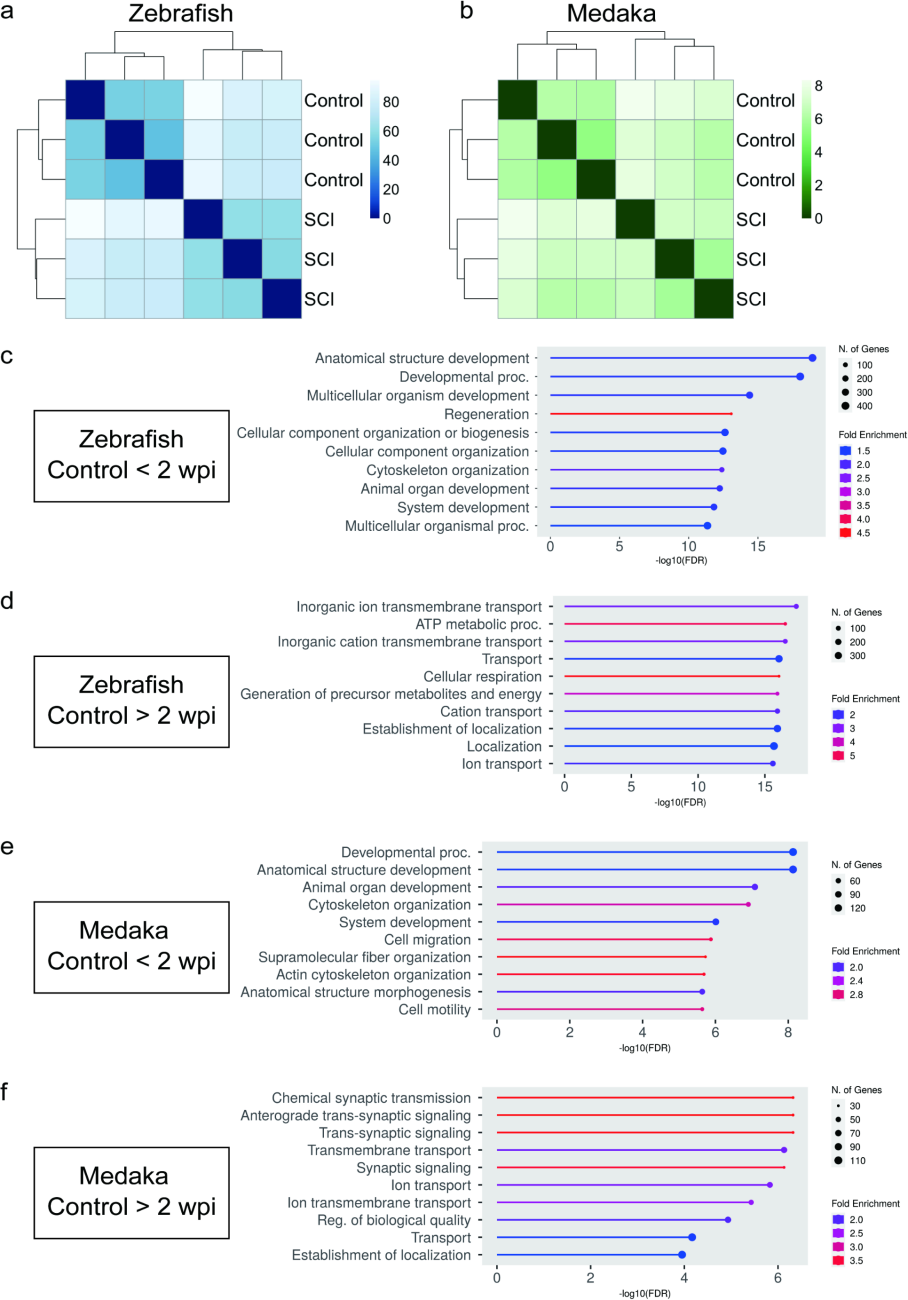
RNA-seq analysis 2 weeks after SCI. (**a**, **b**) Heatmap clustering of samples based on distance matrix. A heatmap of the distance matrix obtained with DESeq2 package to show an overview of similarities and dissimilarities between the RNA-seq samples. The heatmap showed 2 distinct groups of control group and injured group in zebrafish (**a**) and medaka (**b**). The darker the color, the closer the distance between samples. (**c**-**f**) Gene ontology enrichment analysis. The horizontal axes represent − log_10_(FDR). Red color indicates the large fold enrichment, and blue color indicates the small fold enrichment. The size of the circle indicates the number of genes. Top 10 significantly enriched terms of upregulated (**c**, **e**) or downregulated (**d**, **f**) differentially expressed genes after SCI in zebrafish (**c**, **d**) or medaka (**e**, **f**) are shown. FDR, false discovery rate; RNA-seq, RNA sequencing; SCI, spinal cord injury

Clustering analysis revealed that the same group (control or SCI) clustered together in zebrafish (Fig. 4a) and medaka (Fig. 4b), suggesting the reproducibility of the RNA-seq data. Clustering analysis of control group revealed that the same species (zebrafish or medaka) clustered together (Supplementary Fig. 5). We then searched for upregulated (adjusted *P* < 0.05, log_2_ fold change > 0) (Supplementary Table 1a, c) or downregulated genes (adjusted *P* < 0.05, log_2_ fold change < 0) (Supplementary Table 1b, d) after SCI in zebrafish (Supplementary Table 1a, b) and medaka (Supplementary Table 1c, d). To characterize these genes, we performed GO enrichment analysis using ShinyGO. The top five enriched terms in upregulated genes after SCI in zebrafish were Anatomical structure development, Developmental process, Multicellular organism development, Regeneration and Cellular component organization or biogenesis (Fig. 4c, Supplementary Table 2a). The enriched terms in the zebrafish downregulated genes were Inorganic ion transmembrane transport, ATP metabolic process, Inorganic cation transmembrane transport, Transport and Cellular respiration (Fig. 4d, Supplementary Table 2b). In contrast, the terms enriched in the upregulated genes in medaka were Developmental process, Anatomical structure development, Animal organ development, Cytoskeleton organization, and System development (Fig. 4e, Supplementary Table 2c). The terms enriched in the downregulated genes in medaka were Chemical synaptic transmission, Anterograde trans-synaptic signaling, Trans-synaptic signaling, Transmembrane transport, and Synaptic signaling (Fig. 4f, Supplementary Table 2d). In summary, genes upregulated after SCI in zebrafish included those involved in regeneration, whereas genes downregulated after SCI in medaka included those involved in synaptic signaling, which is consistent with their regenerative ability.

**Fig. 5:**
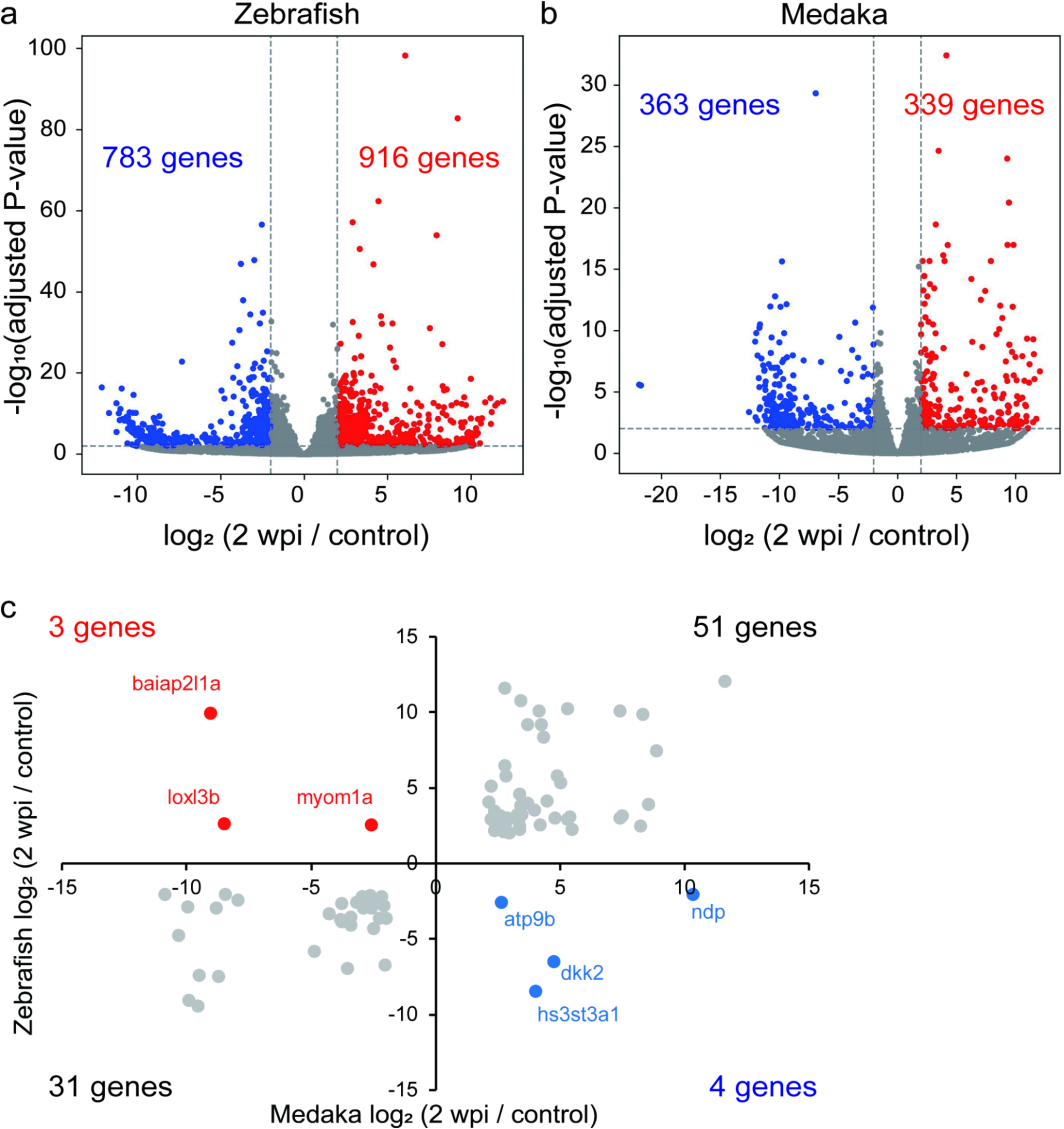
Genes with opposite expression change in zebrafish and medaka identified by RNA-seq. (**a**, **b**) Volcano plots of 2 wpi vs. control in zebrafish (**a**) or medaka (**b**). Vertical axes represent the − log_10_ (adjusted P value), whereas the horizontal axes represent the log_2_ fold-change in expression levels in 2 wpi group compared to those in control group. Red indicates genes that were adjusted *P* < 0.05 (Wald test) and more than 4-fold upregulated. Blue indicates genes that were adjusted *P* < 0.05 (Wald test) and more than 4-fold downregulated. The horizontal dashed lines indicate adjusted *P* = 0.05 and the vertical dashed lines indicate|log_2_ (2 wpi/control)| = 2. (**c**) Genes that are orthologous in zebrafish and medaka. Genes for which the adjusted *P* < 0.05 and|log_2_ fold-change| > 2 were mapped. Horizontal axis represents the log_2_ fold-change in expression levels in 2 wpi group compared to those in control group in medaka, whereas the vertical axis represents the log_2_ fold-change in expression levels in 2 wpi group compared to those in control group in zebrafish. Red indicates genes that were upregulated in zebrafish but downregulated in medaka, whereas blue indicates genes that were downregulated in zebrafish but upregulated in medaka. RNA-seq, RNA sequencing; wpi, weeks post injury

The number of genes that were strongly upregulated (adjusted *P* < 0.05, log_2_ fold change > 2) in injured zebrafish compared to non-injured zebrafish was 916, and the number of genes that were strongly downregulated (adjusted *P* < 0.05, log_2_ fold change < -2) was 783 (Fig. 5a). In medaka, 339 genes were strongly upregulated, and 363 genes were strongly downregulated (Fig. 5b). To identify the genes that showed opposite expression changes between zebrafish and medaka from the genes listed above, we focused on orthologous genes that evolved from a common ancestral gene by speciation and usually retained a similar function in different species (Fig. 5c). Fifty-one genes were upregulated and 31 genes were downregulated in injured zebrafish and medaka compared to non-injured animals. Four genes (*dickkopf WNT signaling pathway inhibitor 2* [*dkk2*], *heparan sulfate-glucosamine 3-sulfotransferase 3A1* [*hs3st3a1*], *ATPase phospholipid transporting 9B* [*atp9b*], *norrin cystine knot growth factor NDP* [*ndp*]) were downregulated in injured zebrafish and upregulated in injured medaka, and three genes (*myomesin 1a* [*myom1a*], *lysyl oxidase-like 3b* [*loxl3b*], *bar/imd domain containing adaptor protein 2 like 1a* [*baiap2l1a*]) were upregulated in injured zebrafish and downregulated in injured medaka.

## Discussion

In this study, we compared the ability of zebrafish and medaka to regenerate the spinal cord using behavioral tests, histological analyses, tracer experiments and gene expression profiles. Our results suggest that functional and histological recovery after SCI is lower in medaka than in zebrafish. To our knowledge, this is the first study to show that medaka have a low capacity to regenerate their spinal cord.

In behavioral tests, the relative speed of the sham group tended to decrease (~ 80%) at 1 wpi (Fig. 1c). This is probably because muscle injury also affects swimming speed. However, the decrease was much less than that of the SCI group (< 50%), and it almost reached the intact level (~ 100%) at 2 wpi or later, suggesting that injury to the spinal cord is the major factor that affects swimming speed in this assay. The functional index of zebrafish in SCI group at 1wpi was approximately 40%. If the spinal cord is transected 4 mm caudal to the brain stem, swimming speed of the zebrafish drops to approximately 20% of the pre-surgery level in a previous report [20]. However, in this study, we transected at the 15th-16th vertebrae level, which is much caudal than the previous study. In zebrafish, a crush injury at the level of 15th-16th vertebrae induces axonal degeneration which mimics human SCI [34], therefore we used this level in this study. The differences in injury level might explain the differences in swimming capacity at 1 wpi. The swimming speed of the zebrafish in the SCI group recovered almost intact level at 4 wpi or later, whereas that of the medaka remained very low. Since both reached a plateau at 4 wpi, it is unlikely that this was due to the slower recovery speed in medaka. Careful interpretation is needed, however, in this cross-species comparison. To analyze them as comparatively as possible, we normalized their speeds and cut the spinal cords at a similar level, but still swimming speed at 1wpi tended to be different between the two species even though we confirmed complete transection in both species in IHC. Because the neural circuits for swimming might differ between the two species, an accurate comparison of functional recovery is difficult. This is especially true for medaka, in which the neural circuits for swimming are less well known, although some neural circuits for swimming and regeneration after SCI have been studied in zebrafish [35]. However, our histological analyses and gene expression profiling partially compensate for this difficulty.

Histological evaluation of glial and neuronal bridging revealed that bridging starts at 2 wpi in both species, and prominent remodeling was observed at 6 wpi in zebrafish, which was consistent with previous studies [20, 33], whereas the thickness of glial or neuronal bridging reached a plateau at 2 or 4 wpi, respectively. The injured tissue was not remodeled to almost intact tissue even at 6 wpi in medaka (Fig. 2). In zebrafish, there is a discrepancy between functional recovery evaluated by the free swimming test, which reached a plateau at 4 wpi, and histological regeneration evaluated by the thickness of both bridges, which continued to recover until at least 6 wpi. A previous study using larval zebrafish also showed cell migration is important for functional recovery at early phase after spinal cord injury followed by neurogenesis, suggesting that dormant neural precursors repair neural circuit at early phase before remodeling [36]. Since we did not directly evaluate the regeneration of the neural circuit responsible for free swimming, the above two evaluations do not necessarily match exactly. Nevertheless, evaluation of the bridging thickness provides information on the gross regenerative ability of the spinal cord, which might also correlate with the regeneration of each neural circuit in the spinal cord; therefore, it has been used as a marker for the regenerative ability of the spinal cord in other studies [12, 37]. Successful remodeling requires coordinated regulation of cell proliferation, differentiation, migration, and neural circuit reorganization. Our results suggest that the molecular mechanisms underlying this process are impaired in medaka.

Tracer experiments revealed that axonal regeneration across the lesion site was unclear in medaka (Fig. 3). In mammals, regeneration of long projecting axons in the central nervous system, such as the corticospinal tract, is very rare [38], in sharp contrast to that in zebrafish [25]. In this study, we did not identify specific tracts, and labeled axons passed 4 mm rostral to the lesion. Although calculation of the regeneration rate of each tract separately is needed to precisely evaluate and compare the regenerative ability of long projecting axons, considering that axons were well-labeled in both species in the intact group, our results suggest that the regenerative ability of long projecting axons in medaka was lower than that in zebrafish. The above three evaluations–free swimming distance, bridging thickness, and regeneration of labeled axons–suggest that the ability to regenerate the spinal cord is lower in medaka than in zebrafish; however, the causal relationship between them needs to be clarified in future studies.

Finally, to identify the factors that might define the differences in the capacity for spinal cord regeneration between zebrafish and medaka, we used RNA-seq to compare gene expression at the SCI site. Through GO enrichment analysis (Fig. 4c-f), we observed that genes related to regeneration were upregulated in zebrafish, which is consistent with previous reports [39–41]. In contrast, in medaka, terms related to regeneration were not included among the top 10 terms upregulated after SCI, and genes related to synaptic signaling were downregulated. This result suggests that the regenerative response starts at 2 wpi, although it was not detected by the evaluation of the bridging thickness. Three genes, growth associated protein 43 (*gap43*), tenascin C (*tnc*), and legumain (*lgmn*), are found among the genes upregulated in zebrafish in the “Regeneration” category. They are also involved in axonal regeneration [39–41]. *gap43* facilitates axonal growth and regeneration by regulating actin dynamics and presynaptic vesicle cycling at axon terminals in rats [42, 43]. F-actin accumulation promoted by GAP43 is important for neurite outgrowth during development and regeneration [44]. Consistent with this, *baiap2l1a*, which encodes a protein that facilitates the formation of F-actin protrusions [45], and *myom1a*, which is related to actin filament binding activity, were also increased in zebrafish but not in medaka in RNA-seq.

Dopamine receptor D3 (*drd3*) was found among the genes downregulated in medaka in “Synaptic signaling” category (Supplementary Table 2d). Dopamine, a neurotransmitter in the central nervous system, is associated with locomotor activity, emotional behavior, and cognitive function [46]. Dysfunction of dopamine transmission is involved in neuropsychiatric diseases, such as depression, and nerve degenerative diseases, such as Parkinson’s disease [46]. In zebrafish, an agonist of the D2 class of dopamine receptors (D2-like: D2, D3, and D4) increases the number of regenerative motor neurons [47], and dopamine is necessary for motor neuron formation. In particular, DRD3 has a high affinity for dopamine [48] and plays an important role in several functions, such as motor activity [46, 49]. It has recently been reported that *drd3* is involved in neuronal development, promoting structural plasticity and neuroprotection [46]. Thus, downregulation of *drd3* in the spinal cord may contribute to the low regenerative ability of medaka. Furthermore, beta-synuclein (*sncb*) was included in the genes downregulated in medaka in the “Synaptic signaling” category (Supplementary Table 2d). SNCB also has a neuroprotective effect [50]; therefore, the downregulation of SNCB in the spinal cord may be involved in the low regenerative ability of medaka. Interestingly, DRD3 forms a heteromeric complex with the β2 subunit of nicotinic acetylcholine receptors, inhibits the accumulation of alpha-synuclein, and induces neuroprotection in dopamine neurons [46]. Additionally, SNCB inhibits the aggregation of alpha-synuclein in vitro [50], and co-expression of SNCB with alpha-synuclein reduces the cytotoxicity of alpha-synuclein [51]. Considering the above reports, *drd3* and *sncb* may also be involved in the reduction of cytotoxicity mediated by alpha-synuclein during spinal cord regeneration. Future functional and histological analyses are required to clarify whether these molecules are involved in the failure of spinal cord regeneration in medaka.

Additionally, we found *dkk2*, which is related to the WNT signaling pathway, was downregulated in zebrafish and upregulated in medaka after SCI in RNA-seq (Fig. 5). WNT signaling promotes spinal cord regeneration in zebrafish [52]. As DKK2 is known to primarily act as a WNT antagonist, *dkk2* might inhibit the *wnt*-mediated regeneration signal in medaka. Interestingly, in adult mice, the expression level of *Dkk2* gradually increases 3 days after SCI [53], suggesting that *Dkk2* might inhibit *Wnt*-mediatad regeneration signals in both mice and medaka. However, DKK2 can also promote WNT signaling in a context-dependent manner [54]. Furthermore, *ndp*, which was downregulated in zebrafish and upregulated in medaka in RNA-seq, activates Wnt/β-catenin signaling through the Frizzled (Fzd) 4 receptor and low-density lipoprotein receptor-related protein 5 [55]. Since low concentrations of *Wnt3* induce outgrowth of retinal axons through Fzd receptors [56], suppression of *ndp* might promote axonal regeneration through maintaining activity of *wnt* signaling at low level. In summary, WNT ligands have concentration-dependent effects on axons, triggering opposing activities depending on the receptor to which they bind [56], therefore upregulation of *dkk2*, which inactivate *wnt* signaling, and downregulation of *ndp*, which activate *wnt* signaling, might keep *wnt* signaling at appropriate level for regeneration. Therefore, careful experiments are needed to correctly understand the role of WNT signaling in spinal cord regeneration.

*Hs3st3a1* was downregulated in zebrafish in RNA-seq. *Hs3st3a1* transfer a 3-O-sulfate group to a glucosamine adjacent to a 2-O-sulfated iduronic acid [57]. 3-O-sulfated heparan sulfate is important for the proliferation of progenitor cells that are dependent on fibroblast growth factor receptor 2b. Therefore, *Hs3st3a1* may be involved in cell proliferation following spinal cord injury in zebrafish.

*Six homeobox 3b (six3b)* was found among the genes upregulated in the “Regeneration” category in zebrafish, but not in medaka, after SCI (Supplementary Table 2). The differentiation potency of Müller glia, mediated by the regulation of TGFβ signaling by *six3b*, is important for retinal regeneration [58]. Similarly, the differentiation potency of ependymoradial glia, a type of radial glia similar to Müller glia, into cells that form glial bridges is important for spinal cord regeneration in zebrafish [12]. Therefore, *six3b* may also be important for the differentiation of ependymoradial glia during spinal cord regeneration in zebrafish. Unlike the successful retinal regeneration in zebrafish, Müller glia generate a limited number of retinal neuronal cell types following retinal injury in medaka [59]. Therefore, the downregulation of *six3b* in the spinal cord suggests that the radial glial differentiation mediated by *six3b* after SCI might be impaired in medaka. Indeed, in this study, IHC demonstrated that glial bridges were thinner in medaka than in zebrafish after SCI (Fig. 2). Therefore, it is possible that the absence of upregulation of *six3b* might affect the remodeling of glial bridges in medaka after SCI.

Interestingly, after spinal cord injury, both zebrafish and medaka showed an increase in genes related to anatomical structure development and developmental processes. However, while zebrafish included genes such as *gap43* and *lgmn*, which are known to be involved in regeneration [39, 40], these genes were not present in medaka. Further comparison of genes in these categories may reveal the differences in the underlying genetics of spinal cord regenerative ability.

This study has several limitations. First, age of fish is not exactly matched between the two species. Although in behavioral test and axonal tracer analysis, the age of fishes is limited in relatively narrow range, in IHC and RNA-seq analyses, the range is relatively broad. Since age might also affect the regeneration capacity, it is important to analyze age dependent effect on regenerative capacity in both species in the future study.

Second, possibility that medaka has delayed regenerative ability is not excluded. Our analysis is limited to 8 weeks after injury. Considering that functional indices reaches plateau at 4wpi, the possibility of delayed functional recovery is relatively low, but whether it reaches plateau or not is not clear in IHC. Analyses in much later points such as 12 weeks or 16 weeks after injury would reveal whether medaka has delayed regenerative ability or is missing the regenerative ability even in the later phases. However, present study at least shows that medaka’s regenerative ability is lower than that of zebrafish at early phases.

Third, variation of swimming speed is relatively high in the behavioral test. Functional indices were significantly different between zebrafish sham and medaka sham group at 3 and 6 wpi, but considering the values were similar in both group in other time points, this might be due to high variation. The fact that even though medaka sham and SCI groups are randomly allocated littermates, raw swimming speed was significantly higher in medaka SCI group compared to medaka sham group before surgery also reflects high variation. Although the difference of functional indices between medaka SCI and zebrafish SCI is very clear and the conclusion is unlikely wrong in this behavioral test, additional more sophisticated behavioral tests might lead more solid conclusion in future studies.

Forth, quantification for axonal tracing experiment was not conducted in this study. Although our whole mount imaging is effective to show gross morphology of regenerated axons, it was difficult to set consistent image acquisition parameters mainly due to curvature of the tissue, which makes quantitative evaluation difficult. Detailed neuroanatomical analyses are needed in the future studies.

Fifth, gene expression profiling was conducted at only 2wpi. Considering that spinal cord regeneration is a dynamic phenomenon and many factors change in each phase, gene expression profiling at many time points after spinal cord injury is important to understand difference between medaka and zebrafish completely in future studies.

Sixth, translational expression is not examined in this study. Since translational expression does not necessarily correspond to transcriptional expression, examination of translational expression by experiments such as mass spectrometry or western blotting is important to expand our knowledge in future study.

Seventh, functional analyses are not conducted in this study. To show the function of target genes we discussed above, loss- or gain-of-function analyses and rescue experiments are needed in the future studies.

In conclusion, zebrafish and medaka have different spinal cord regeneration abilities and genetic profiles. This study shows that comparison of the spinal cord regeneration abilities of zebrafish and medaka could be a promising research field to elucidate new factors that determine spinal cord regeneration ability.

## Electronic Supplementary Material

Below is the link to the electronic supplementary material.

Supplementary Material 1: Supplementary Figure 1

Supplementary Material 2: Supplementary Figure 2

Supplementary Material 3: Supplementary Figure 3

Supplementary Material 4: Supplementary Figure 4

Supplementary Material 5: Supplementary Figure 5

Supplementary Material 6: Supplementary Movie 1

Supplementary Material 7: Supplementary Movie 2

Supplementary Material 8: Supplementary Movie 3

Supplementary Material 9: Supplementary Movie 4

Supplementary Material 10: Supplementary Movie 5

Supplementary Material 11: Supplementary Movie 6

Supplementary Material 12: Supplementary Table 1

Supplementary Material 13: Supplementary Table 2

Supplementary Material 14: Supplementary Table 3

Supplementary Material 15: Supplementary File Captions

## Data Availability

The RNA-seq data were deposited in the DNA Data Bank of Japan (accession number: PRJDB18466).
