# Supplementary figures and images for: Comparison of Spinal Cord Regeneration Capacity in Zebrafish and Medaka
Source: Neurochem Res. 2025 Apr 25;50(3):153. doi: 10.1007/s11064-025-04389-9 (PMC12031921; doi:10.1007/s11064-025-04389-9)

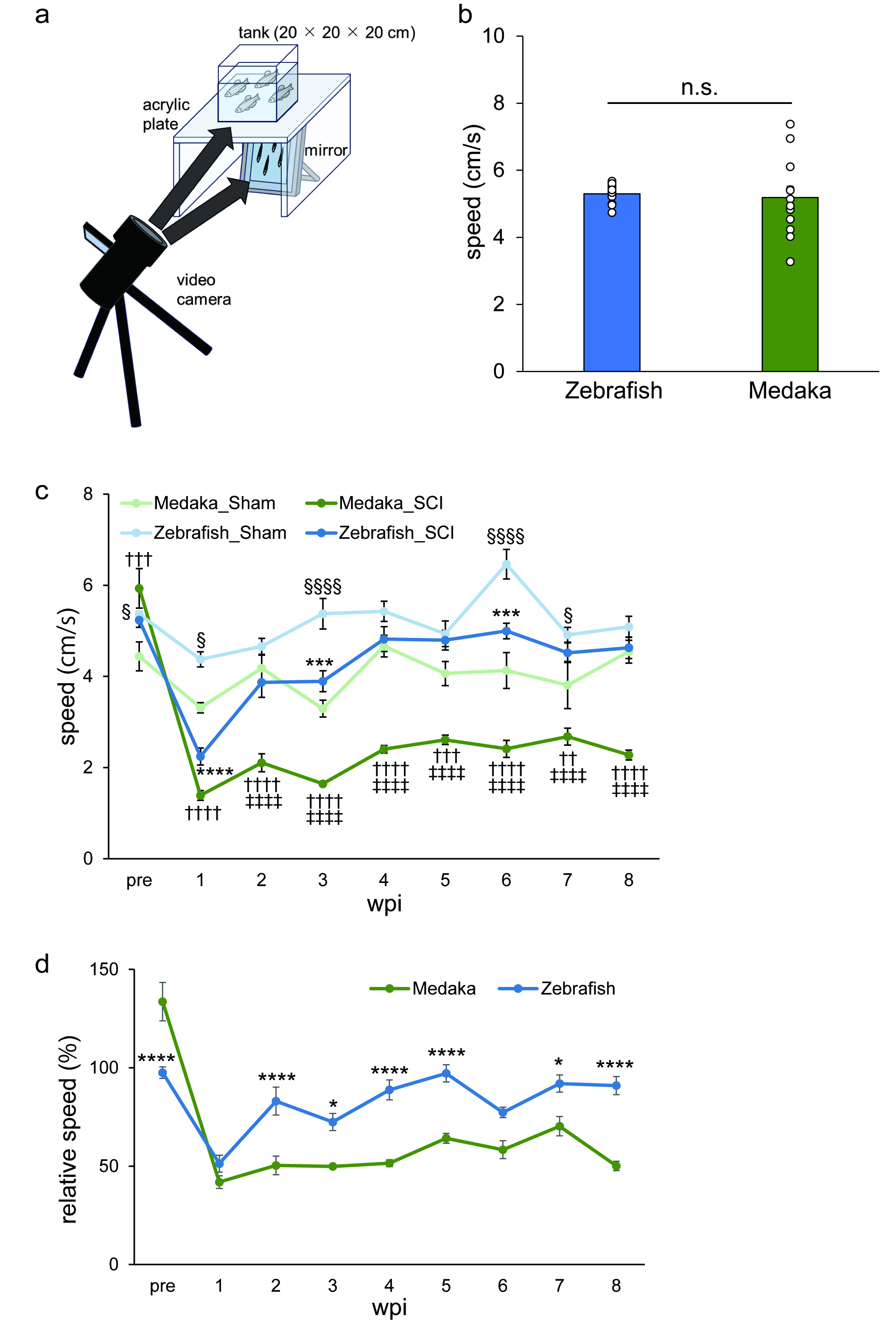

Supplement: Supplementary file 1 — Supplementary Material 1: Supplementary Figure 1 [file 11064_2025_4389_MOESM1_ESM.tif]

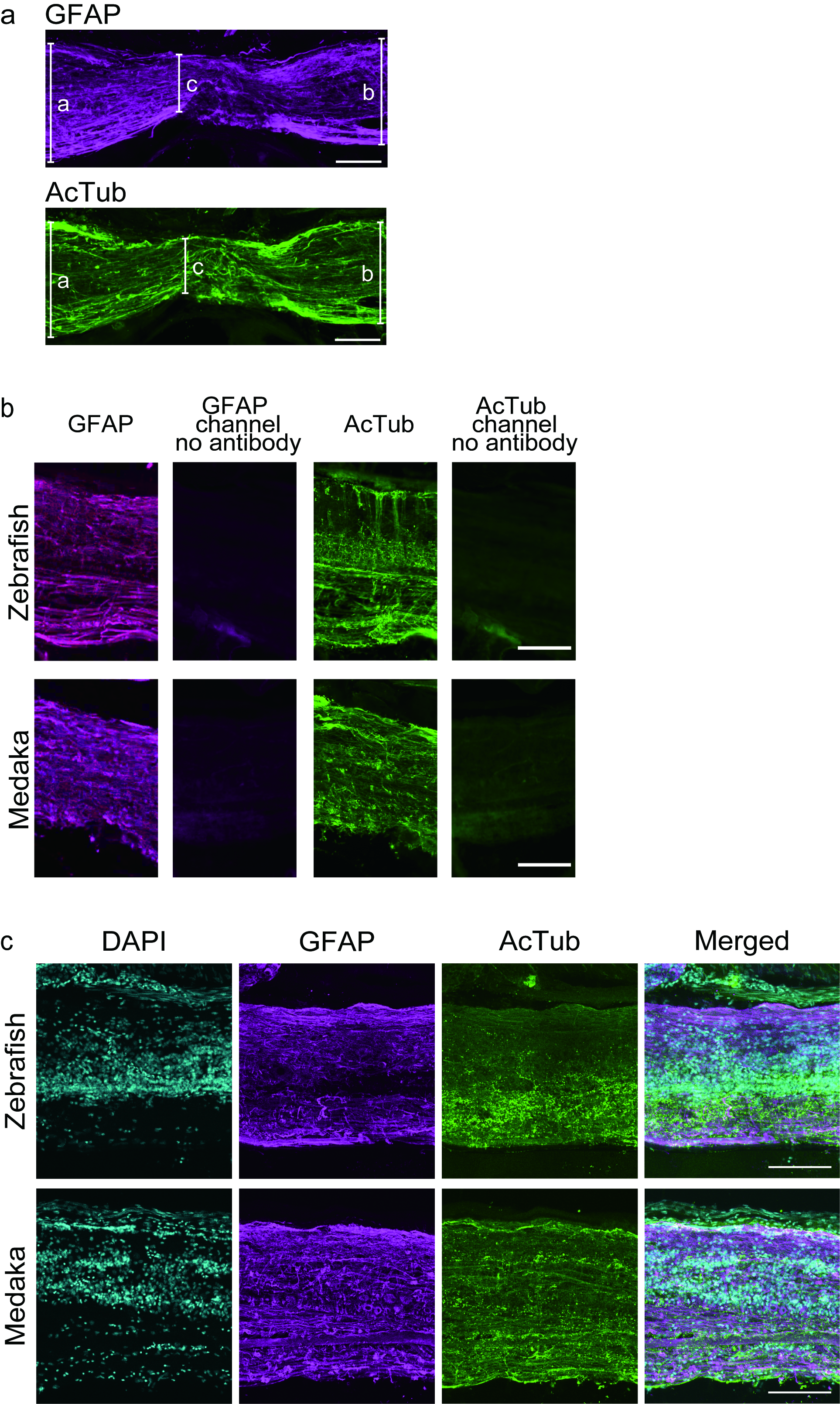

Supplement: Supplementary file 2 — Supplementary Material 2: Supplementary Figure 2 [file 11064_2025_4389_MOESM2_ESM.tif]

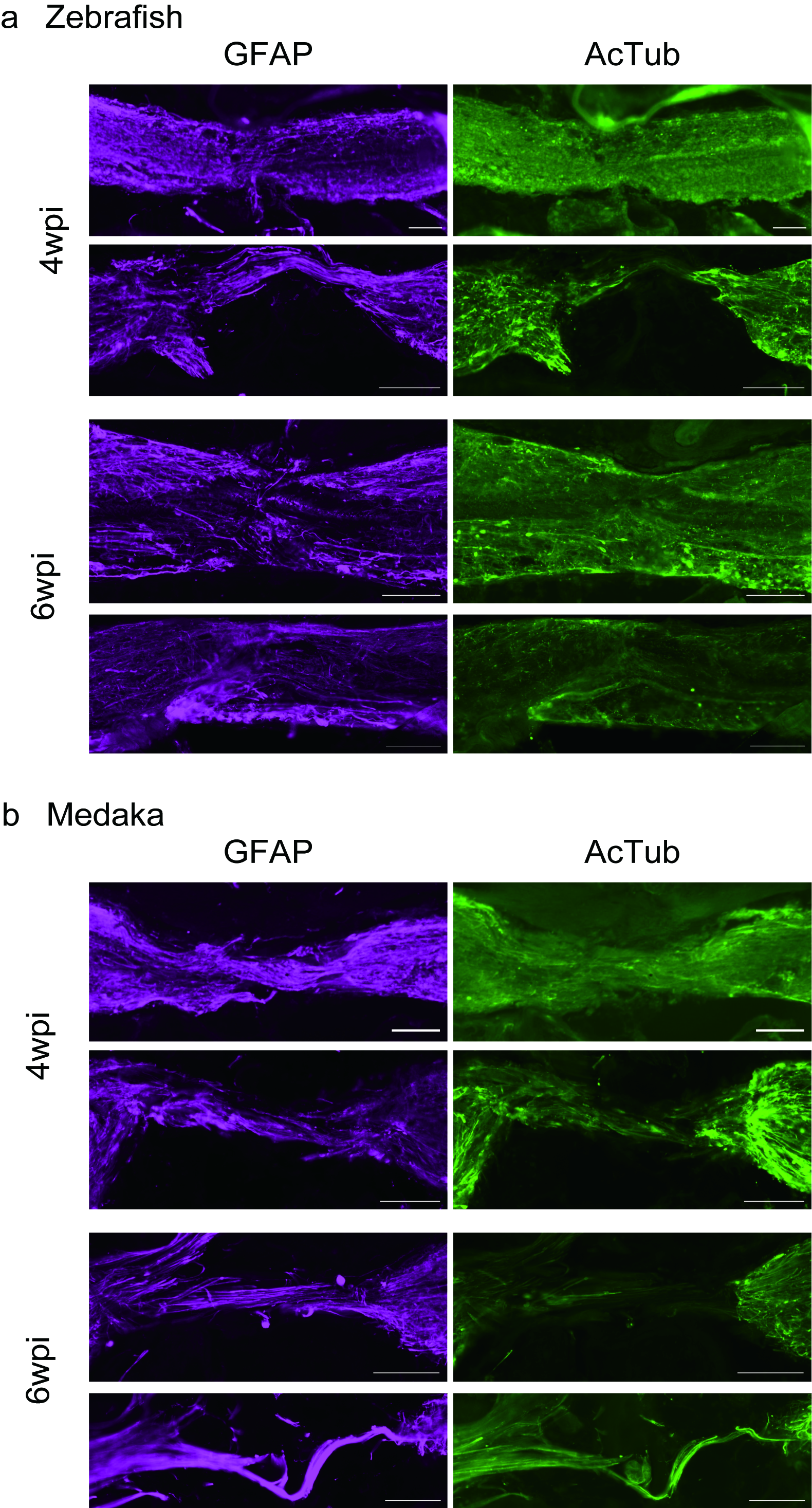

Supplement: Supplementary file 3 — Supplementary Material 3: Supplementary Figure 3 [file 11064_2025_4389_MOESM3_ESM.tif]

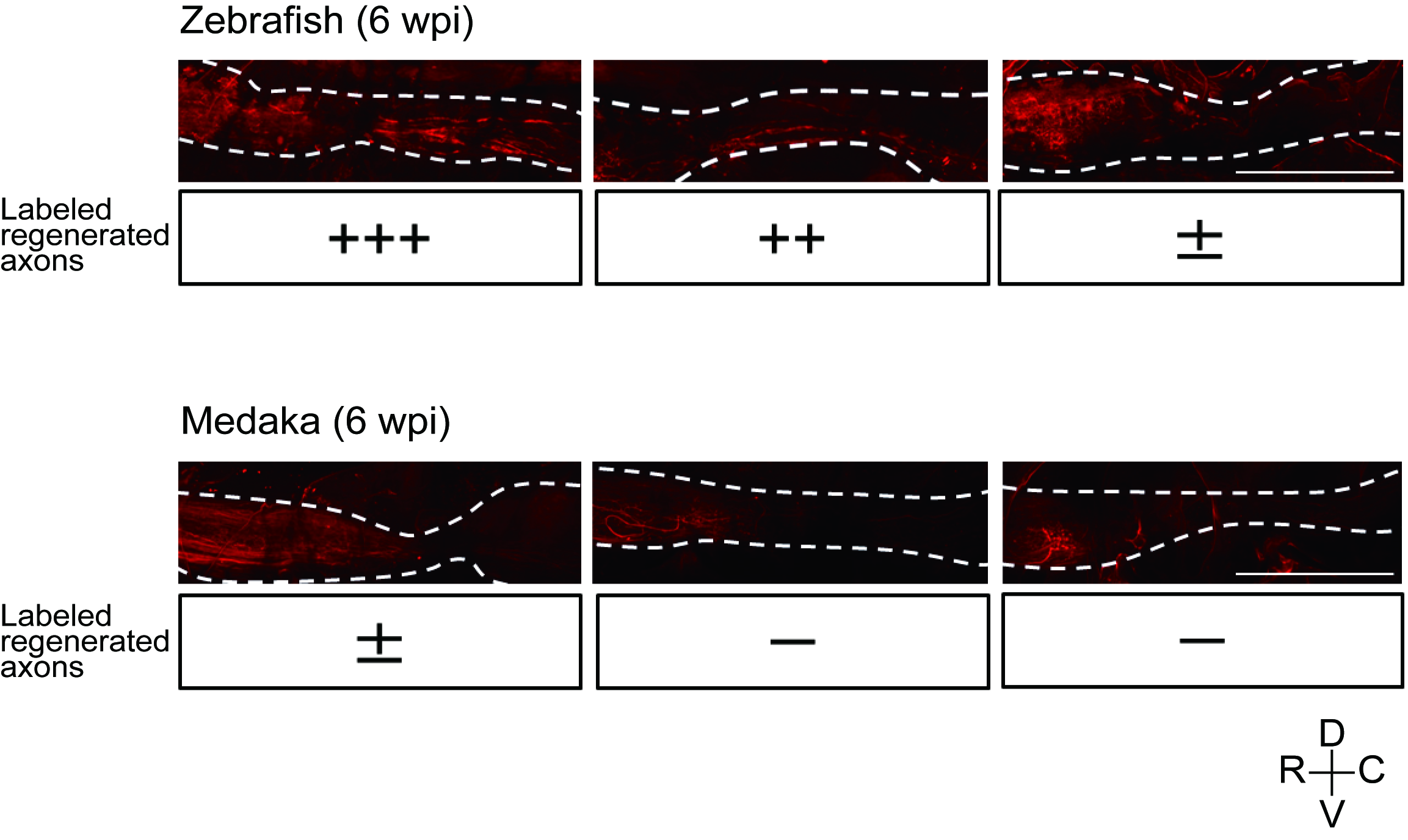

Supplement: Supplementary file 4 — Supplementary Material 4: Supplementary Figure 4 [file 11064_2025_4389_MOESM4_ESM.tif]

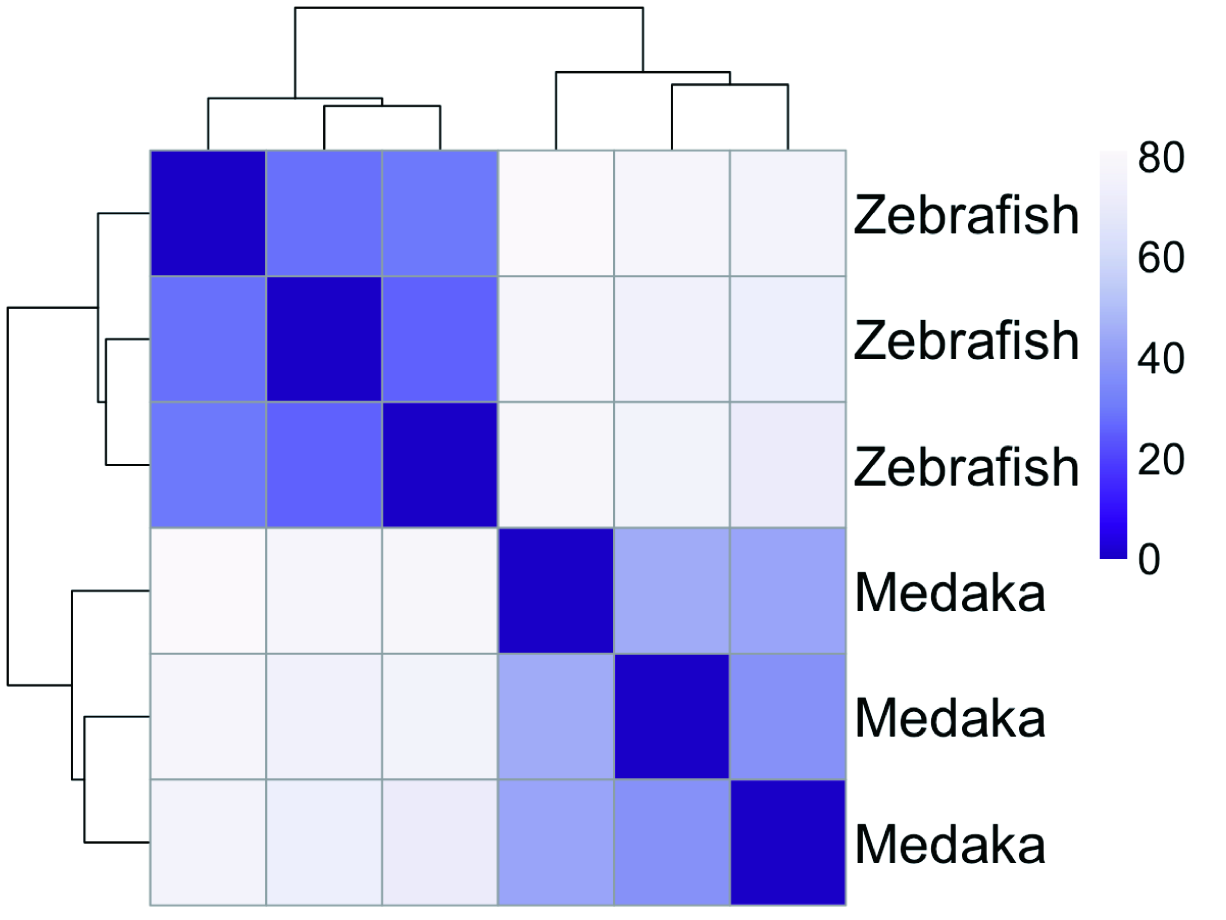

Supplement: Supplementary file 5 — Supplementary Material 5: Supplementary Figure 5 [file 11064_2025_4389_MOESM5_ESM.tif]
