# Supplementary material for: Comparison of Spinal Cord Regeneration Capacity in Zebrafish and Medaka
Source: Neurochem Res. 2025 Apr 25;50(3):153. doi: 10.1007/s11064-025-04389-9 (PMC12031921; doi:10.1007/s11064-025-04389-9)
Supplement: Supplementary file 15 — Supplementary Material 15: Supplementary File Captions [file 11064_2025_4389_MOESM15_ESM.docx]

**Supplementary File Captions**

**Supplementary Fig. 1** The overview of the behavioral test.

(a) Overview of experimental setting for 3D swimming tracking of fish. A schematic diagram to show the 3D swimming assay apparatus. (b) speed before surgery in behavioral test. Blue indicates zebrafish, green indicates medaka. Mean ± SEM. n = 12 (Student’s t-test). (c) Average speed in behavioral test. Light green indicates medaka with sham surgery, dark green indicates medaka with SCI, light blue indicates zebrafish with sham surgery, and dark blue indicates zebrafish with SCI. The vertical axis represents the speeds. The horizontal axis represents the wpi. The statistical results of the main comparisons are presented. The statistical results for comparisons between all groups are provided in Supplementary Table 3. Mean ± SEM. n = 5-6, *** P < 0.001, **** P < 0.0001 (vs. zebrafish with Sham surgery), ††P < 0.01, †††P < 0.001, ††††P < 0.0001 (vs. medaka with Sham surgery), ‡‡‡‡P < 0.0001 (vs. zebrafish with SCI), §P < 0.05, §§§§P < 0.0001 (vs. medaka with Sham surgery), two-way ANOVA with Sidak’s multiple comparisons test. P < 0.0001 (time), P < 0.0001 (condition) P < 0.0001 (interaction). (d) Average speed in behavioral test. Green indicates medaka, blue indicates zebrafish. The vertical axis represents the relative speed, with the value of the speed of sham group set as 100％. The horizontal axis represents the wpi. Mean ± SEM. n = 5-6, * P < 0.05, **** P < 0.0001, two-way ANOVA with Sidak’s multiple comparisons test. P < 0.0001 (time), P < 0.0001 (condition) P < 0.0001 (interaction), SCI, spinal cord injury; SEM, standard error of the mean; wpi, weeks post injury

**Supplementary Fig. 2** Histological evaluation of spinal cord regeneration.

(a) Dorsoventral distances from the rostral (a) or caudal (b) spinal cord to the lesion or at the thinnest part of the lesion (c) were measured in sagittal sections. Regeneration rate = c/((a + b)/2). Top: GFAP; bottom: AcTubs. (b) Sagittal sections of intact zebrafish (top) or medaka (bottom). No antibody indicates a negative control in which no primary antibody was added. (c) Sagittal sections of intact zebrafish (top) or medaka (bottom), compared to the site of spinal cord injury in other fish. AcTub, acetylated tubulin; DAPI, 4′,6-diamidino-2-phenylindole; GFAP, glial fibrillary acidic protein. Scale bars: 100 µm

**Supplementary Fig. 3** Immunohistological comparison between zebrafish and medaka at 4 wpi and 6 wpi.

(a, b) Sagittal section of lesion site labeled with anti-GFAP or anti-AcTub antibody in zebrafish (a) or medaka (b) at 4, 6 wpi are shown. Scale bars: 100 µm

**Supplementary Fig. 4** Comparison of axonal extension in zebrafish and medaka at 6 wpi.

Labeled regenerated axons indicate the level of axonal regeneration across the injury site.

+++ : Many extended axons are visible

++ : A moderate number of extended axons are visible

± : A few axons with weak fluorescence are visible

－: No extended axons are visible

D, dorsal; V, ventral; R, rostral; C, Caudal. Scale bar: 500 µm

**Supplementary Fig. 5** Heatmap clustering of samples based on distance matrix between intact zebrafish and medaka.

**Supplementary Table 1**

Differentially expressed genes after SCI in zebrafish and medaka

(a) Genes upregulated after SCI in zebrafish (adjusted P < 0.05, log_2_ fold change > 0). (b) Genes downregulated after SCI in zebrafish (adjusted P < 0.05, log_2_ fold change < 0). (c) Genes upregulated after SCI in medaka (adjusted P < 0.05, log_2_ fold change > 0). (d) Genes downregulated after SCI in medaka (adjusted P < 0.05, log_2_ fold change < 0).

genes, ensembl gene ID; baseMean, the average of the normalized count values, divided by size factors, taken over all samples; log2FoldChange, the effect size estimate. This value, which is reported on a logarithmic scale to base 2, indicates how much the gene expression seems to have changed between the control and 2 wpi groups; lfcSE, the standard error estimate for the log2 fold change estimate; stat, the value of the test statistic for the genes; pvalue, P-value of the test for the gene; padj, adjusted P-value for multiple testing for the genes

**Supplementary Table 2**

Genes included in each top 10 GO term in GO enrichment analysis

(a) Genes upregulated after SCI in zebrafish (adjusted P < 0.05, log_2_ fold change > 0). (b) Genes downregulated after SCI in zebrafish (adjusted P < 0.05, log_2_ fold change < 0). (c) Genes upregulated after SCI in medaka (adjusted P < 0.05, log_2_ fold change > 0. (d) Genes downregulated after SCI in medaka (adjusted P < 0.05, log_2_ fold change < 0)

**Supplementary Table 3**

Statistical results of Fig. 1c and Supplementary Fig. 1c

(a) Statistical results of the comparison of the functional index between zebrafish and medaka in Fig. 1c. (b) Statistical results of the comparison of the speed between zebrafish and medaka in Supplementary Fig. 1c.

**Supplementary Movie 1**

Video for the behavioral test. Zebrafish at 0 wpi

**Supplementary Movie 2**

Video for the behavioral test. Zebrafish at 1 wpi

**Supplementary Movie 3**

Video for the behavioral test. Zebrafish at 4 wpi

**Supplementary Movie 4**

Video for the behavioral test. Medaka at 0 wpi

**Supplementary Movie 5**

Video for the behavioral test. Medaka at 1 wpi

**Supplementary Movie 6**

Video for the behavioral test. Medaka at 4 wpi
